# Academic careers in global pulmonary and critical care medicine

**DOI:** 10.7189/jogh.10.010313

**Published:** 2020-06

**Authors:** Alfred Papali, Janet V Diaz, E Jane Carter, Juliana C Ferreira, Rob Fowler, Tewodros H Gebremariam, Stephen B Gordon, Burton W Lee, Srinivas Murthy, Elisabeth D Riviello, T Eoin West, Neill KJ Adhikari

**Affiliations:** 1 Division of Pulmonary & Critical Care Medicine, Atrium Health, Charlotte, North Carolina, USA; 2 Division of Pulmonary & Critical Care Medicine, University of North Carolina School of Medicine, Chapel Hill, North Carolina, USA; 3 World Health Organization, Geneva, Switzerland; 4 Department of Medicine, Alpert School of Medicine, Brown University, Providence, Rhode Island, USA; 5 Divisão de Pneumologia, Instituto do Coração, Hospital das Clínicas, Faculdade de Medicina, Universidade de São Paulo, São Paulo (SP), Brasil; 6 Department of Critical Care Medicine, Sunnybrook Health Sciences Centre and Interdepartmental Division of Critical Care Medicine and Institute of Health Policy, Management, and Evaluation, University of Toronto, Toronto, Ontario, Canada; 7 Addis Ababa University College of Health Sciences, Addis Ababa, Ethiopia; 8 The Malawi Liverpool Wellcome Trust Clinical Research Programme, Queen Elizabeth Central Hospital, Blantyre, Malawi; 9 Liverpool School of Tropical Medicine, Liverpool, UK; 10 University of Pittsburgh School of Medicine, Pittsburgh, Pennsylvania, USA; 11 Department of Paediatrics, University of British Columbia, Vancouver, British Columbia, Canada; 12 Beth Israel Deaconess Medical Center and Harvard Medical School, Boston, Massachusetts, USA; 13 Division of Pulmonary, Critical Care and Sleep Medicine, Department of Medicine, University of Washington, Seattle, Washington, USA

The burden of respiratory and critical illness is high worldwide, yet specialist care is underrepresented in low- and middle-income countries (LMICs) [1]. For many areas of medicine, the past decade has witnessed tremendous growth in global health opportunities for trainees; however, these opportunities tend to be restricted to individual institutions and geographic regions and academic global pulmonary and critical care medicine (PCCM) remains a relatively novel concept [2]. Consequently, PCCM fellows and junior faculty at institutions with limited global health mentorship have little guidance in building successful global health careers.

This paper highlights various pathways to develop a successful academic career in PCCM and global health. Ranging from traditional academic medicine to private practice, professional societies to transnational health policy bodies, the challenges of balancing international work with clinical and other professional demands are discussed in **Table 1** provides examples of and links to specific opportunities. A more comprehensive discussion with personal anecdotes and advice from current global PCCM faculty can be found separately (publication pending, *Journal of Global Health Reports*).

**Table 1:** Career options in academic global pulmonary and critical care medicine

Pathway	Examples	Locations	Links
Professional societies	ATS-MECOR	USA	https://www.thoracic.org/about/global-public-health/mecor-program/
	ESICM global intensive care working group	International	https://www.esicm.org/resources/thematics/ global -intensive-care-2/
World Health Organization	IMAI	Global	http://www.who.int/careers/en/
Physician-scientist global health training	Johns Hopkins University	USA	http://www.globalncd.org/global-health-track/
	Malawi-Liverpool-Wellcome Trust Clinical Research Programme	Malawi/UK	https://www.mlw.mw/
Faith-based/NGO	Kijabe Hospital	Kenya	http://kijabehospital.org/blog/eccco-program
	St. Luke Foundation	Haiti	http://www.stlukehaiti.org/health-care
Educational partnerships	East Africa Training Initiative (EATI)	Ethiopia	https://eatiethiopia.org/
	REACH	Haiti	http://haecc.org
Medical ethics	MERETI	Middle East	http://www.mereti-network.net/
Pediatrics	WFPICCS	Global	https://www.wfpiccs.org
	PECC-Kenya	Kenya	http://www.pecc-kenya.org

NGO – non-governmental organization, ATS – American Thoracic Society, MECOR – Methods in Epidemiological, Clinical and Operations Research, ESICM – European Society of Intensive Care Medicine, IMAI – Integrated Management of Adult Illness, REACH – Research and Education consortium for Acute Care in Haiti, MERETI – Middle East Research Training Initiative, WFPICCS – World Federation of Pediatric and Intensive Care Societies, PECC – Pediatric Emergency and Critical Care

## CAPACITY-BUILDING

Building both clinical and research capacity in LMICs is paramount to generate locally relevant health research that informs practice, policy and population health. The importance of training the next generation of young clinicians and investigators in LMICs with adequate mentoring is increasingly recognized [3]. Two examples include the East Africa Training Initiative (EATI) and the American Thoracic Society (ATS) Methods in Epidemiologic, Clinical, and Operations Research (MECOR) Program.

EATI, a PCCM training program in Addis Ababa, is a partnership between Ethiopian physician leadership and American academic medical centers that began in 2013. It is creating a cadre of well-trained physicians through a two-year intensive fellowship program focusing on clinical, research, advocacy and telemedicine development [4]. International faculty have a continuous presence, with each faculty member being on site for a month at a time.

MECOR is a capacity-building course designed to help PCCM clinicians, academicians, and public health professionals conduct research relevant to LMICs. The program organizes courses in multiple global regions annually and boasts >1800 graduates over 25 years [5]. International and local faculty volunteers teach the three week-long courses and provide distance mentoring. Thus, faculty have the opportunity to get involved in education and research in LMICs with either very short trips or from their home institution.

## WORLD HEALTH ORGANIZATION (WHO)

WHO addresses health policy by focusing on underlying social, environmental and economic determinants of health and developing evidence-based guidance. Its engagement is of particular relevance in LMICs with insufficient clinical, health policy, and research expertise. Areas of activity relevant to PCCM abound as evolving patterns of global burden of disease have increased attention to acute care [6] and non-communicable illness. Focus areas include pulmonary infection treatment, tobacco-related mortality reduction, air quality improvement, road safety initiatives and emergency and essential surgical care. The WHO increasingly views critical care as an essential component given its substantial role in recent outbreaks.

Other than in outbreaks, WHO generally does not employ clinicians to carry out direct patient care. Clinical expertise, multilingualism and geographic mobility are valued skills for full-time professional positions. Temporary consultant positions for education and training, especially in LMIC environments with limited capacity, are a common WHO role. Variable-duration unpaid internships provide practical experience for trainees, and certain countries sponsor “Junior Professional Officer” positions to provide early-career young professionals with practical experience in multilateral technical co-operation.

**Figure:**
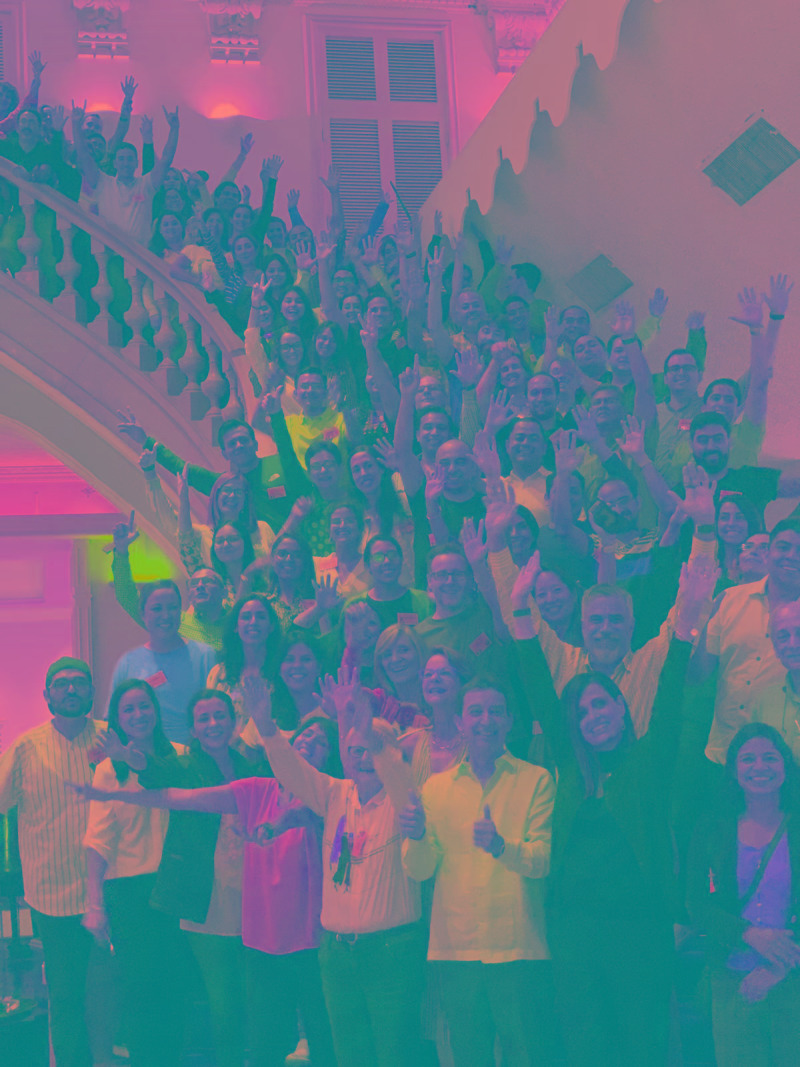
Photo: ATS MECOR Latin America Level 1 group photo, Mexico, 2019 (from the collection of Juliana Ferreira, Sao Paulo, Brazil; used with permission).

## PHYSICIAN-SCIENTIST PATHWAY FOR GLOBAL HEALTH

Research in LMICs should be of the highest quality, clinically relevant to local health needs, and emphasize capacity development. Because less research is performed in LMICs, whatever is done needs to be done very well. Grounding research in local health needs can optimize clinical utility and lead to clinical questions of greater scope with the potential to improve outcomes for a larger population. Researchers in LMICs, where few PCCM training programs exist, have an obligation to develop the next generation of researchers and policy-makers who can implement research results.

For PCCM trainees aiming to become independent physician-scientists focused on LMICs, there are several considerations. First, global health can take many forms, and while a substantial time commitment in the field is essential to long-term impact, important contributions from analysis of global data sets are possible [7]. Video conferencing and social networking can facilitate international collaboration. Second, for fellowship training, select an institution with success in nurturing independent physician-scientists. Equally important is identifying a qualified and supportive mentoring team and acquisition of specific training in global health knowledge, leadership, and research ethics. Third, trainees should learn to be productive academically. Grant-writing skills, first author abstracts and manuscripts, training grants and career development awards are key. Protected research time is short and precious, so crafting a timeline and realistic goals early-on is essential.

## FAITH-BASED HEALTH CARE ORGANIZATIONS

Faith-based health care organizations (FBH) account for a substantial part of health care in many LMICs since virtually all major religions encourage caring for the poor and the vulnerable in society [8]. There is substantial variation in activities of FBHs globally, but one example relevant to PCCM is the Emergency Critical Care Clinical Officer program (ECCCO) Program at Kijabe Hospital (Kenya), which is owned and operated by the Africa Inland Church. ECCCO is an 18-month training program for physicians, nurse anesthetists and clinical officers to provide long-term, sustainable, quality acute care. Many trainees and faculty from HIC academic institutions have volunteered as clinical mentors.

FBH work is not for everyone. One should explore fully the institution’s practices to ensure comfort with faith traditions, since philosophies and practices can vary widely. FBHs are not immune to issues of limited human and material resources, high attrition of workers, and poor governance that plague many hospitals. Nevertheless, FBH work can be rich and rewarding for those comfortable in this environment.

## PRIVATE PRACTICE

Finding the balance between clinical work and global health can be challenging in an academic environment. An alternative is to split work between private or community-based practice and global health pursuits. Part-time private practice may offer sufficient salary support to offset non-clinical time and may provide the flexibility for overseas activities. However, this approach may be more suitable for inpatient practice and may challenge personal and family development. Feasibility is likely to be contingent on supportive features of the HIC private practice setting, since demands of each practice can vary widely. For individuals with such flexibility, the global health private practice model may prove to be an attractive “road less traveled.”

## PEDIATRIC CONSIDERATIONS

All of these career paths are applicable to pediatric PCCM. International societies, such as the World Federation of Pediatric and Intensive Care Societies, have made global training opportunities and capacity building a focus, given the tremendous global needs. Additionally, several training programs in North America have robust institutional linkages based on established global health centers in many children’s hospitals. Training programs for pediatric critical and emergency care have begun (for example, in Kenya) and are starting to scale up in other regions. Opportunities for advocacy, education, research, and clinical service abound, and interested early-career individuals can access these opportunities through any of these pathways.

## CONCLUSION

There is no single pathway to a successful career in academic global PCCM. Specialist clinical skills, public health, research and teaching are all needed in government, international public health, non-governmental organization (NGO) and faith-based settings. Determination and creativity during training are crucial and should feed into strategic thinking and a 5-year individual development plan, which can help to articulate specific goals and career strategies [9]. Participating in global health-focused professional society opportunities also provides face-to-face networking opportunities between trainees and potential mentors, who can provide career advice and potentially offer opportunities to join existing projects [10]. Trainees and junior faculty interested in academic global PCCM will find innumerable obstacles to developing this non-traditional career pathway, particularly in institutions where global health is less emphasized. However, the strategies outlined herein offer diverse and feasible pathways for success.
